# Ultrasonic evaluation of muscle functional recovery following free functioning gracilis transfer, a preliminary study

**DOI:** 10.1186/s40001-020-00473-8

**Published:** 2021-02-05

**Authors:** Yi Hou, Jiantao Yang, Bengang Qin, Liqiang Gu, Jia Zheng

**Affiliations:** 1 Department of Orthopedics, Henan Provincial People’s Hospital, No. 7, Weiwu Road, Zhengzhou, 450003 China; 2 Department of Microsurgery and Orthopedic Trauma, the First Affiliated Hospital of Sun Yat-Sen University, 58 Second Zhongshan Road, Guangzhou, 510080 China

**Keywords:** Ultrasound, Muscle contraction, Functional muscle transplantation, Muscle strength

## Abstract

**Background:**

Ultrasonic measurement has not been utilized to assess the functional recovery of transplanted muscle. This study aimed to investigate the feasibility of using B-ultrasound measurement to assess muscle recovery following free functioning gracilis transfer.

**Methods:**

From January 2009 to January 2014, 35 patients receiving free functioning gracilis transfer to treat total brachial plexus injury were enrolled. B-ultrasound was adopted to determine the cross-sectional area (CSA) of transplanted gracilis muscle at rest and contraction state. The ratio of pre- to post-transplant CSA value at rest state was defined as muscle bulk ratio (MBR). The ratio of CSA value at contraction state to rest state was defined as contraction ratio (CR).

**Results:**

Patients with muscle strength *M* ≥ 4 had significantly higher CR1 (post-transplant), CR2 (pre-transplant), and range of motion (ROM, joint mobility) than those with muscle strength *M* < 4. The CR1 > CR2 group had significantly higher CR1, muscle strength, and ROM than the CR1 ≤ CR2 group. The MBR > 1 group had significantly higher muscle strength than the MBR ≤ 1 group. CR1 value was highly correlated with muscle strength and with ROM. CR2 value was moderately correlated with muscle strength and ROM. Multivariate linear regression analysis showed that a higher CR1/CR2 value was associated with a higher muscle strength and joint mobility. The CR1 > CR2 group had better muscle strength and ROM than the CR1 ≤ CR2 groups.

**Conclusion:**

B-ultrasound measurement can quantitatively reflect muscle strength following gracilis transfer, and CR value could be a potential indicator for functional recovery of the transplanted gracilis muscle.

**Level of Evidence:** Prognostic studies, Level II.

## Background

Free functioning gracilis transfer is a surgical procedure for limb motor function in patients with Brachial plexus avulsion, brachial plexus nerve injuries, traumatic muscle loss, facial paralysis, and tumor resection [1–8]. It transplants gracilis muscles with vascular nerve pedicles to the upper limbs, suturing the affected blood vessels and motor nerves to replace the function of lost muscles in the affected area [9]. The reinnervation after gracilis muscle transfer is a slow process. Based on the recovery condition, auxiliary surgery (such as fusion of wrist joint) is usually required to promote functional recovery [10, 11]. Therefore, timely assessing functional recovery of transplanted muscle can provide a reference for subsequent decision-making.

Currently, assessment methods for functional recovery of transplanted muscle include electromyography [12], manual muscle test (MMT) [13], and magnetic resonance imaging (MRI) [14]. However, all these evaluation methods have some defects. EMG results are not always consistent with muscle strength recovery of the transplanted gracilis muscle. MMT is easy to conduct but subjective, and muscle strength M4 cannot be quantitatively assessed. MRI cannot dynamically assess muscle contraction function, and it is expensive.

B-ultrasound is non-invasive, convenient, and cheap, and can reflect muscle strength by echo density, muscle thickness, cross-sectional area, and intermuscular volume [15–19]. Compared to the static observation of MRI, B-ultrasound can observe the dynamic contraction of muscle. Ultrasonic measurement can easily obtain morphological parameters of muscle, such as muscle thickness and cross-sectional area. The cross-sectional area measured by B-ultrasound has been used to investigate the effect of resistance training on muscle strength [16, 20]. Since muscle morphological parameters are closely related to muscle function, muscle strength and function can be reflected by morphological parameters in ultrasonic measurement [20]. The correlation between ultrasonic morphologic parameters and muscle strength has been demonstrated [16, 21, 22]. However, B-ultrasound has not been used to determine functional recovery of transplanted muscle following. In this study, therefore, we aimed to investigate the feasibility of B-ultrasound measurement for evaluation of muscle recovery following free functioning gracilis transfer.

## Methods

### Participants

From January 2009 to January 2014, 35 patients receiving free functioning gracilis transfer to treat total brachial plexus injury in our hospital were enrolled. All patients received free functioning gracilis transfer for the first time. This study was approved by the institutional review board of our hospital. Written informed consent was obtained from each patient.

### Surgical procedure

All patients underwent free functioning gracilis transfer to reconstruct the functions of elbow flexion–finger/thumb extension, and the ipsilateral accessory nerve was used as a donor motor nerve. The reconstruction of the transplanted gracilis muscle was located from the lateral clavicle to the extensor carpi radialis longus muscle and the tendon of extensor pollicis longus muscle at the dorsal side of the distal forearm. The gracilis muscle was completely harvested from the origin on the pubic ramus to the pes anserine tendon around the interior knee joint, which length can meet the needs of reconstruction (Additional file 1: Figure S1). Patient's elbow was flexed in 90°, and the gracilis muscle was placed on the shoulder-upper arm-anterolateral elbow-the dorsal side of the middle 1/3 of the forearm. The proximal end of the gracilis muscle was fixed to the acromion or outer clavicle periosteum, spanning the elbow joint, and the distal end was sutured to extensor digitorum communis and extensor pollicis longus tendons with appropriate tension. The elbow joint was maintained at functional position by plaster cast. Therefore, the gracilis muscle can be used for long-distance reconstruction without tendon grafts, and the functions of elbow flexion, finger extension, and thumb extension can be simultaneously reconstructed (Additional file 2: Figure S2). If necessary, the other gracilis muscle can be placed in the forearm to reconstruct the function of extrinsic muscles of the hand. The purpose of double gracilis transplantation was to restore the grasping function of the hand to a greater extent.

### Muscle strength measurement and range of motion (ROM)

The muscle strength was assessed by manual muscle strength test and the ROM of elbow flexion was determined by a protractor tool. All assessment was performed by a trained physician who was not involved in the ultrasonic measurement. To avoid observation bias, the definition of M4 strength was modified to “muscle can resist at least the examiner’s one finger or at least 1 kg in weight” according to Lin et al.’s study [23].

### Ultrasonic measurement and outcome analysis

All B-ultrasound examinations are performed using the Venue 40 Ultrasound (GE Healthcare, USA) and the cross-sectional area (CSA) of the with gracilis muscle was obtained based on the ultrasound images with the Scion Image software (National Institutes of Health, Bethesda MD). All the process was performed by the same physician and can be completed within 10 min. Before and after transplantation, the ultrasound examinations and the corresponding CSA of transplanted gracilis muscle were measured at rest state and contraction state. Each ultrasound examination was continuously repeated five times and the corresponding CSA value was averaged.

The origin and midpoint of the abdominal muscle were determined with B-ultrasound. Before transplantation, the CSA was measured at the midpoint of the abdominal muscle. Before transplantation, the CSA of the gracilis muscle at rest state was measured at the neutral position of the hip joint, while the CSA of the gracilis muscle at the contraction state was determined at 30° abduction of the hip joint.

For CSA measurement after transplantation, the patient was placed in the supine position, and the upper extremity was straightened. The midpoint of the transplanted gracilis muscle was determined by B-ultrasound scan, and the plane was used to measure the CSA of the gracilis muscle at rest state. The CSA of the gracilis muscle at the contraction state was determined in the isometric contraction state with shoulder abduction of 20° and elbow flexion at 60°, which was the posture used to restore the original length of the transplanted muscle during surgery. Restoring the original length of the transplanted muscle in this position will not cause the elbow joint straightening obstacle, and can achieve the best elbow flexion–finger extension effect. Therefore, patients received B-ultrasound examination in the same posture to make the pre-transplant and post-transplant results comparable.

### Muscle bulk ratio (MBR) and contraction ratio (CR)

**Fig. 1:**
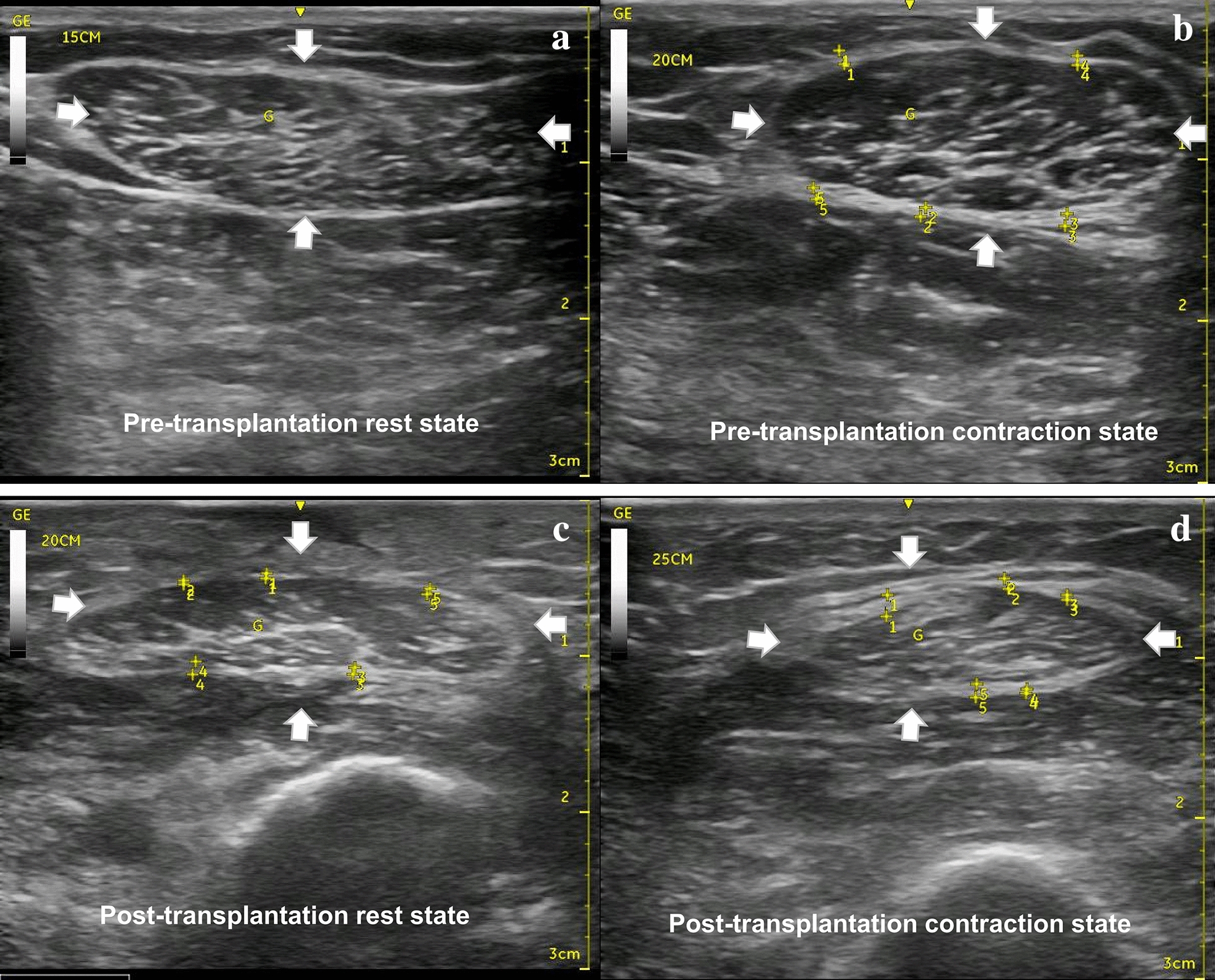
Ultrasound image of cross-sectional area of the gracilis muscle. The white arrows indicate muscle cross-section area (CSA). **a** Pre-transplantation rest state; **b** Pre-transplantation contraction state; **c** post-transplantation rest state; **d** post-transplantation contraction state. The ratio of pre- to post-transplant CSA value of gracilis muscle at rest state was defined as muscle bulk ratio (MBR). The ratio of CSA value at the contraction state to that at rest state was defined as contraction ratio (CR). The CR values of gracilis muscle before and after transplantation were defined as CR-2 and CR-1, respectively. MBR = CSA in **c**/CSA in **a**; CR1 = CSA in **d**/CSA in **c**; CR2 = CSA in **b**/CSA in **a**

The ratio of pre- to post-transplant CSA value of gracilis muscle at rest state was defined as MBR. The ratio of contraction state to rest state CSA value was defined as contraction ratio (CR). The CR values of gracilis muscle before and after transplantation were defined as CR-2 and CR-1, respectively (Fig. 1). The patients were divided into following dichotomous groups for subgroup analysis of muscle strength or ROM: the MBR > 1 vs. MBR < 1; CR-1 > CR-2 group vs. CR-1 < CR-2 group; and muscle strength ≥ M4 vs. muscle strength < M4 groups.

### Statistical analysis

Continuous variables are presented as mean ± standard deviation (SD), while categorical data are shown as number and percentage (%). Student’s independent t-test was used to compare the differences between groups. If normality of continuous variables was not assumed, non-parametric analysis Mann–Whitney U test would be used instead. Chi-square test and Fisher’s exact test (if any expected value lower than 5 was observed) were used for categorical data. Pearson’s correlation coefficient was used to report the correlation among continuous variables. Univariate and multivariate linear regression models were used to investigate the associations between CR/MBR and muscle strength/range of motion (ROM). A *P*-value lower than 0.05 would be recognized as significance. All analyses were performed using IBM SPSS Version 20 (SPSS Statistics V20, IBM Corporation, Somers, New York).

## Results

### Patient’s clinical characteristics

A total of 35 patients (32 males and 3 females, mean age = 30.40 ± 8.40 years) undergoing free functioning gracilis transfer were included. The mean follow-up period of all patient’s was 19.11 ± 6.51 (range 8 to 32) months after transplantation. Table 1 demonstrates demographic and clinical characteristics, including muscle strength (M), CR1 (post-transplant), CR2 (pre-transplant), MBR, and ROM. The detailed ultrasonic measurement outcomes and muscle strength of all 35 patients are summarized in Additional file 3: Table S1.

**Table 1:** Patient’s clinical characteristics

Parameters	Mean ± SD / *N* (%)
Gender	
Male	32 (91.43)
Female	3 (8.57)
Age, years	30.40 ± 8.40
Follow-up period, month	19.11 ± 6.51
Muscle strength (M)	3.26 ± 0.89
Muscle strength group	
* M* < 4	17 (48.57)
* M* ≤ 4	18 (51.43)
CR1	1.23 ± 0.15
CR2	1.22 ± 0.13
Relation between CR1 and CR2	
CR1 > CR2	20 (57.14)
CR1 ≤ CR2	15 (42.86)
MBR	1.00 ± 0.14
MBR group	
> 1	18 (51.43)
≤ 1	17 (48.57)
ROM	73.66 ± 27.39

*CR* contraction ratio, *MBR* muscle bulk ratio, *ROM* range of motion

CR1 was the post-transplant CR value, while CR2 was the pre- transplant CR value

### Subgroup analysis stratified by muscle strength, relative CR, and MBR

The ultrasonic measurement outcomes and muscle strength were compared by the following dichotomous groups, including muscle strength (*M* < 4 vs. *M* ≥ 4), CR value order (CR1 > CR2 vs. CR1 ≤ CR2), and MBR (MBR > 1 vs. MBR ≤ 1). It was found that the *M* ≥ 4 group had significantly higher CR1, CR2, and ROM values than the M < 4 group (all *P* < 0.001, Table 2). The CR1 > CR2 group had significantly higher CR1, muscle strength, and ROM than the CR1 ≤ CR2 group (all *P* < 0.01, Table 3). The MBR > 1 group had significantly higher muscle strength than the MBR ≤ 1 group (*P* = 0.038, Table 4).

**Table 2:** Comparisons between muscle strength groups

Parameters	*M* < 4	*M* ≥ 4	*P*
CR1	1.10 ± 0.06	1.35 ± 0.10	< 0.001
Muscle strength	2.47 ± 0.62	4.00 ± 0.00	< 0.001
ROM	52.12 ± 22.22	94.00 ± 11.54	< 0.001
CR2	1.15 ± 0.09	1.30 ± 0.12	< 0.001
MBR	1.02 ± 0.18	0.99 ± 0.09	0.576

*CR* contraction ratio, *MBR* muscle bulk ratio, *ROM* range of motion

CR1 was the post-transplant CR value, while CR2 was the pre- transplant CR value

**Table 3:** Comparisons between comparative relations of CR groups

Parameters	CR1 > CR2	CR1 ≤ CR2	*P*
CR1	1.29 ± 0.15	1.14 ± 0.11	0.002
Muscle strength	3.60 ± 0.68	2.80 ± 0.94	0.006
ROM	85.20 ± 22.40	58.27 ± 26.39	0.003
CR2	1.21 ± 0.12	1.24 ± 0.15	0.483
MBR	0.98 ± 0.11	1.04 ± 0.17	0.237

*CR* contraction ratio, *MBR* muscle bulk ratio, *ROM* range of motion

CR1 was the post-transplant CR value, while CR2 was the pre- transplant CR value

**Table 4:** Comparisons between MBR groups

Parameters	MBR > 1	MBR ≤ 1	*P*
CR1	1.24 ± 0.14	1.21 ± 0.17	0.660
Muscle strength	3.56 ± 0.51	2.94 ± 1.09	0.038
ROM	78.67 ± 16.98	68.35 ± 35.06	0.272
CR2	1.24 ± 0.11	1.21 ± 0.15	0.500
MBR	1.11 ± 0.11	0.89 ± 0.05	< 0.001

*CR* contraction ratio, *MBR* muscle bulk ratio, *ROM* range of motion

CR1 was the post-transplant CR value, while CR2 was the pre- transplant CR value

### Associations between CR/MBR and M/ROM

To further evaluate the potential of ultrasonic outcomes as the indicator for muscle strength, correlation analysis between CR/MBR and muscle strength/ROM was performed. As shown in Table 5, CR1 had the highest correlation coefficient with muscle strength (0.808) and ROM (0.847) (both *P* < 0.01); CR2 had a medium correlation coefficient with M (0.491) and ROM (0.556) (both *P* < 0.01). However, MBR had no significant correlation with muscle strength and ROM (both *P* > 0.05). The coefficient between M and ROM was 0.903 (*P* < 0.001).

**Table 5:** Correlation coefficient results

Parameters	CR1	CR2	MBR
Muscle strength	0.808**	0.491**	0.204
ROM	0.847**	0.556**	0.017

*CR* contraction ratio, *MBR* muscle bulk ratio; *ROM* range of motion

CR1 was the post-transplant CR value, while CR2 was the pre- transplant CR value

**P < 0.001

The associations between CR/MBR and M/ROM were further evaluated by univariate and multivariate linear regression analyses. Multivariate linear regression analysis adjusted for patient’s age and gender showed that a higher CR1/CR2 value suggested a higher muscle strength and ROM value (all *P* < 0.01, Table 6). Meanwhile, the CR1 > CR2 group had a better muscle strength and ROM than the CR1 ≤ CR2 groups (all *P* < 0.01, Table 6). However, there were no significant results in the regression models of MBR (all *P* > 0.05).

**Table 6:** Univariate and multivariate linear regression results

	Univariate		Multivariate^1^	
Parameters	*B*^2^	*P*	*B*	*P*
Muscle strength				
CR1	4.70 (3.49–5.91)	< 0.001	4.61 (3.37–5.84)	< 0.001
CR2	3.35 (1.24–5.45)	0.003	3.47 (1.35–5.59)	0.002
Relation between CR1 and CR2				
CR1 > CR2	ref	–	ref	–
CR1 ≤ CR2	− 0.80 (− 1.36 to 0.24)	0.006	− 0.80 (− 1.39 to 0.21)	0.010
MBR	1.30 (− 0.90 to 3.51)	0.239	1.50 (-0.75–3.74)	0.183
ROM				
CR1	152.31 (118.49–186.13)	< 0.001	150.44 (115.59–185.29)	< 0.001
CR2	117.16 (55.06–179.27)	< 0.001	122.02 (59.24–184.79)	< 0.001
Relation between CR1 and CR2				
CR1 > CR2	ref	–	ref	–
CR1 ≤ CR2	− 26.93 (− 43.73 to 10.14)	0.003	− 27.34 (− 45.41 to 9.27)	0.004
MBR	3.26 (− 66.37 to 72.90)	0.925	7.87 (− 64.01 to 79.74)	0.825

*CR* contraction ratio, *MBR* muscle bulk ratio, *ROM* range of motion

CR1 was the post-transplant CR value, while CR2 was the pre- transplant CR value

^1^ Multivariate results were adjusted with patient’s age and gender

^2^ *B* stands for regression coefficient

## Discussion

In this study, we investigated the feasibility of B-ultrasound to evaluate the muscle recovery following free functioning gracilis transfer. The results showed that The *M* ≥ 4 group had significantly higher CR1, CR2, and ROM value (joint mobility) as compared with the *M* < 4 group. The CR1 > CR2 group had significantly higher CR1, muscle strength, and ROM than the CR1 ≤ CR2 group. The MBR > 1 group had significantly higher muscle strength than the MBR ≤ 1 group. CR1 value was highly correlated with muscle strength (*r* = 0.808) and ROM (*r* = 0.847), while CR2 value was moderately correlated with muscle strength (*r* = 0.491) and ROM (*r* = 0.556). Multivariate linear regression analysis showed that a higher CR1/CR2 value was associated with a higher muscle strength and higher joint mobility. Meanwhile, the CR1 > CR2 group had better muscle strength and ROM than the CR1 ≤ CR2 groups (all *P* < 0.01). Taken together, these results suggested that B-ultrasound measurement can quantitatively reflect muscle function following gracilis transfer, and CR could be a potential indicator for muscle function recovery.

Currently, ultrasonic parameters which could be used as the indicator for muscle contraction include echo intensity (EI) [21], muscle thickness [24], muscle fiber pennation [25], and measures of muscle architecture [19]. EI reflects muscle function by the density of high echo signals in the muscle but is susceptible to be affected by the connective tissue between the subcutaneous and muscle bundles. A study on the changes in muscle strength by Jacobs et al. has confirmed that EI is not an optimal indicator of muscle strength [21]. In addition, the transplanted gracilis muscle undergoes denervation and fibrosis; therefore, EI is not suitable for function evaluation following free functioning gracilis transfer. Muscle fiber pennation reflects the muscle contraction by the angle between the direction of muscle fibers and the long axis of the muscle.[26] Gracilis muscle is a non-feathery muscle, and could not be measured by muscle fiber pennation. The transplanted muscle is close to the skins, and the ultrasonic test is susceptible to be affected by probe-induced pressure; hence, muscle thickness measurement is also not applicable for muscle transplantation.

The gracilis muscle is non-feathery muscle, and its muscle fibers are arranged in the same direction as the tendon. During the muscle contraction, all the muscle fibers slide in parallel. Therefore, the histological cross-sectional area of the muscle fibers of the gracilis muscle is substantially the same as the gross anatomical cross-sectional area [19]. Therefore, B-ultrasound can be used to measure CSA, and the dynamic contraction of muscles can be reflected by CSA changes (i.e., CR) at rest and contraction. The transplanted gracilis muscle undergoes denervation and nerve re-innervation, and the muscle volume changes during this process. The MBR and CR are standardized indicators calculated based on CSA, which can eliminate the impact of muscle volume change during denervation and nerve re-innervation [27]. Hence, we chose CR and MBR as the indicators to explore the recovery of gracilis muscle functional after free functioning gracilis transfer.

Our results showed a high correlation between CR value and muscle function indexes, indicating that CR can be used to dynamically evaluate the recovery of muscle function after transplantation. In addition, muscle strength and ROM were significantly higher in the CR1 > CR2 group than in the CR1 ≤ CR2 group, suggesting that patient with elevated muscle CR after transplantation has a better recovery of muscle strength and joint mobility. We observed an increase in the CR value of the gracilis muscle after transplantation. One of the possible reasons is as follows: since the motion range of elbow bowing is larger than hip adduction, the muscle fibers of transplanted gracilis muscle need to be parallel sliding for a longer distance in the elbow bowing. Therefore, the CR value was elevated after transplantation. Our results showed that the mean CR1 values of the muscle strength ≥ *M*4 group and the CR1 > CR2 group were 1.35 ± 0.10 and 1.29 ± 0.15, respectively. Based on these results, we propose that CR1 of 1.3 might be an important reference value for transplanted muscle recovery. When the CR value of transplanted muscle reaches 1.3, the muscle might have satisfactory recovery. However, this reference value should be further validated in a large trial.

In this study, we also evaluated MBR, which reflects muscle volume change, i.e., atrophy (MBR < 1) or hypertrophy (MBR > 1). We found that patients who had no atrophy with gracilis muscle (MBR > 1) showed no significant advantage in muscle strength and joint mobility. Subgroup analysis of muscle strength also showed that MBR value was not significantly different between two muscle strength groups. Correlation analysis also revealed that there was no correlation between MBR and muscle strength/joint mobility. All these results suggested that MBR cannot be used as an indicator for muscle recovery following free functioning gracilis transfer.

Our preliminary findings demonstrated the feasibility of B-ultrasound for assessing functional recovery after gracilis transfer. Early muscle contraction changes may not be easily detected by physical examination. B-ultrasound examination can dynamically detect the progress of muscle contraction recovery, as well as the tendon-gliding function. When a lower postoperative CR value is found in early postoperative period, the patients can receive physical therapy to promote nerve reinnervation. In the late postoperative period (e.g., at 1 year after surgery, there is no progress in muscle strength for 3 consecutive months), a second femoral muscle transplantation could be performed to rebuild the flexor. Even though this was a prospective study, the sample size was relatively small. In the future, a large trial should be conducted to validate the findings of this study.

## Conclusions

In summary, our findings demonstrated that B-ultrasound measurement can quantitatively reflect muscle function following gracilis transfer, and CR value could be a potential indicator for muscle function recovery. The CR value of transplanted muscle was highly correlated with the muscle function, which can be used to dynamically assess muscle recovery after muscle transplantation. Elevated CR value of gracilis muscle after transplantation suggested a better prognosis.

## Supplementary Information

**Additional file 1: Figure S1.** Design of functional free gracilis muscle transfer. The gracilis muscle iscompletely harvested from the origin on the pubic ramus to the pes anserine tendon around theinterior knee joint.

**Additional file 2: Figure S2.** (A) Elbow flexion and fingers and thumb extension reconstruction; (B). Fingers and thumb reconstruction.

**Additional file 3: Table S1.** Ultrasound and follow-up results of all 35 patients

## Data Availability

All the data and materials have been presented in the main paper.

## Abbreviations

MMT: Manual muscle test
MRI: Magnetic resonance imaging
ROM: Range of motion
CSA: Cross-sectional area
MBR: Muscle bulk ratio
CR: Contraction ratio
