# Supplementary figures and images for: Ultrasonic evaluation of muscle functional recovery following free functioning gracilis transfer, a preliminary study
Source: Eur J Med Res. 2021 Feb 5;26:17. doi: 10.1186/s40001-020-00473-8 (PMC7863516; doi:10.1186/s40001-020-00473-8)

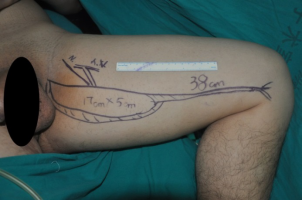

Supplement: Supplementary file 1 — Additional file 1: Figure S1. Design of functional free gracilis muscle transfer. The gracilis muscle iscompletely harvested from the origin on the pubic ramus to the pes anserine tendon around theinterior knee joint. [file 40001_2020_473_MOESM1_ESM.tif]
