# Supplementary material for: Ultrasonic evaluation of muscle functional recovery following free functioning gracilis transfer, a preliminary study
Source: Eur J Med Res. 2021 Feb 5;26:17. doi: 10.1186/s40001-020-00473-8 (PMC7863516; doi:10.1186/s40001-020-00473-8)
Supplement: Supplementary file 2 — Additional file 2: Figure S2. (A) Elbow flexion and fingers and thumb extension reconstruction; (B). Fingers and thumb reconstruction. [file 40001_2020_473_MOESM2_ESM.doc]

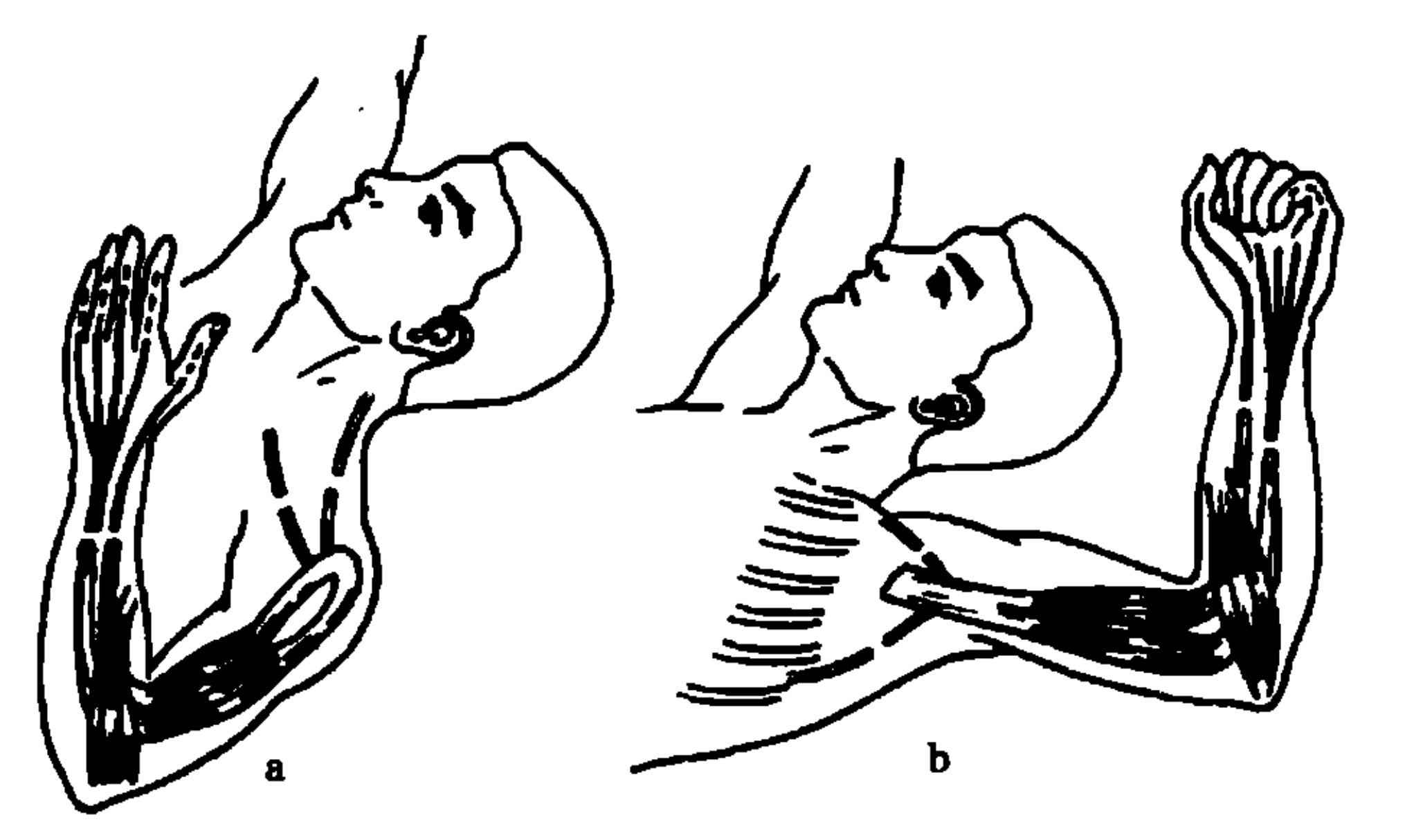


Supplementary Figure 2. (A) Elbow flexion and fingers and thumb extension reconstruction; (B). Fingers and thumb reconstruction.
