# Supplementary material for: Ultrasonic evaluation of muscle functional recovery following free functioning gracilis transfer, a preliminary study
Source: Eur J Med Res. 2021 Feb 5;26:17. doi: 10.1186/s40001-020-00473-8 (PMC7863516; doi:10.1186/s40001-020-00473-8)
Supplement: Supplementary file 3 — Additional file 3: Table S1. Ultrasound and follow-up results of all 35 patients [file 40001_2020_473_MOESM3_ESM.docx]

**Supplementary Table S1. Ultrasound and follow-up results of all 35 patients**

|  |  |  |  | After transplantation | | |  |  | Before transplantation | | |  |
| --- | --- | --- | --- | --- | --- | --- | --- | --- | --- | --- | --- | --- |
| No | Age | Gender | Follow-up period, month | Contraction CSA | Rest CSA | CR1 | Muscle strength | ROM | Contraction CSA | Rest CSA | CR2 | MBR |
| 1 | 32 | M | 24 | 105.93 | 88.45 | 1.20 | 4 | 86 | 94.34 | 81.12 | 1.16 | 1.09 |
| 2 | 21 | M | 30 | 61.98 | 56.77 | 1.09 | 3 | 82 | 57.34 | 54.13 | 1.06 | 1.05 |
| 3 | 22 | F | 18 | 60.73 | 48.64 | 1.25 | 4 | 94 | 63.35 | 54.56 | 1.16 | 0.89 |
| 4 | 17 | M | 14 | 46.34 | 34.77 | 1.33 | 4 | 100 | 49.64 | 39.65 | 1.25 | 0.88 |
| 5 | 26 | M | 16 | 143.37 | 112.64 | 1.27 | 4 | 90 | 140.44 | 119.43 | 1.18 | 0.94 |
| 6 | 22 | M | 23 | 58.34 | 46.19 | 1.26 | 4 | 92 | 59.34 | 44.00 | 1.35 | 1.05 |
| 7 | 24 | M | 8 | 52.06 | 42.26 | 1.23 | 3 | 84 | 55.47 | 48.63 | 1.14 | 0.87 |
| 8 | 46 | M | 32 | 89.48 | 61.61 | 1.45 | 4 | 106 | 97.68 | 71.85 | 1.36 | 0.86 |
| 9 | 27 | M | 23 | 45.80 | 33.06 | 1.39 | 4 | 110 | 56.65 | 35.98 | 1.57 | 0.92 |
| 10 | 39 | M | 24 | 102.71 | 71.74 | 1.43 | 4 | 104 | 92.57 | 69.75 | 1.33 | 1.03 |
| 11 | 43 | F | 24 | 67.02 | 64.64 | 1.04 | 2 | 30 | 69.38 | 68.24 | 1.02 | 0.95 |
| 12 | 29 | M | 18 | 94.90 | 63.22 | 1.50 | 4 | 110 | 86.75 | 59.24 | 1.46 | 1.07 |
| 13 | 22 | M | 18 | 102.52 | 65.55 | 1.56 | 4 | 100 | 105.57 | 79.56 | 1.33 | 0.82 |
| 14 | 22 | M | 14 | 68.45 | 65.48 | 1.05 | 1 | 0 | 93.56 | 74.79 | 1.25 | 0.88 |
| 15 | 37 | M | 22 | 31.67 | 31.35 | 1.01 | 2 | 34 | 41.65 | 39.89 | 1.04 | 0.79 |
| 16 | 32 | M | 24 | 115.40 | 104.84 | 1.10 | 3 | 60 | 114.73 | 105.33 | 1.09 | 1.23 |
| 17 | 34 | M | 20 | 54.91 | 53.55 | 1.03 | 3 | 64 | 48.67 | 40.76 | 1.19 | 1.31 |
| 18 | 21 | M | 28 | 69.68 | 63.48 | 1.10 | 2 | 50 | 79.46 | 69.79 | 1.14 | 0.91 |
| 19 | 33 | M | 18 | 74.58 | 56.26 | 1.33 | 4 | 90 | 69.47 | 55.74 | 1.25 | 1.01 |
| 20 | 28 | M | 8 | 62.39 | 52.71 | 1.18 | 3 | 72 | 58.56 | 50.67 | 1.16 | 1.04 |
| 21 | 31 | M | 27 | 67.68 | 60.77 | 1.11 | 3 | 60 | 59.99 | 48.34 | 1.24 | 1.26 |
| 22 | 25 | M | 14 | 62.67 | 50.11 | 1.25 | 4 | 86 | 60.31 | 53.56 | 1.13 | 0.94 |
| 23 | 32 | M | 14 | 77.74 | 63.55 | 1.22 | 4 | 68 | 68.84 | 54.69 | 1.26 | 1.16 |
| 24 | 46 | M | 30 | 98.13 | 92.90 | 1.06 | 2 | 40 | 108.84 | 102.75 | 1.06 | 0.90 |
| 25 | 35 | M | 12 | 44.85 | 39.16 | 1.15 | 3 | 70 | 42.68 | 36.86 | 1.16 | 1.06 |
| 26 | 24 | M | 8 | 69.42 | 60.84 | 1.14 | 3 | 64 | 76.45 | 67.22 | 1.14 | 1.06 |
| 27 | 29 | M | 12 | 51.16 | 39.71 | 1.29 | 4 | 76 | 56.48 | 38.23 | 1.48 | 1.04 |
| 28 | 51 | M | 22 | 45.48 | 40.84 | 1.11 | 2 | 68 | 63.76 | 45.67 | 1.40 | 0.89 |
| 29 | 26 | M | 18 | 41.87 | 39.29 | 1.07 | 2 | 30 | 52.85 | 49.68 | 1.06 | 0.79 |
| 30 | 35 | M | 10 | 66.32 | 63.10 | 1.05 | 2 | 32 | 78.46 | 65.56 | 1.20 | 0.96 |
| 31 | 34 | M | 12 | 62.84 | 55.35 | 1.14 | 3 | 46 | 65.46 | 55.89 | 1.17 | 1.35 |
| 32 | 45 | M | 24 | 169.54 | 117.03 | 1.45 | 4 | 88 | 134.78 | 108.56 | 1.24 | 1.08 |
| 33 | 26 | F | 20 | 77.41 | 54.35 | 1.42 | 4 | 108 | 72.56 | 56.78 | 1.28 | 0.96 |
| 34 | 25 | M | 20 | 70.06 | 52.06 | 1.35 | 4 | 96 | 64.68 | 50.33 | 1.29 | 1.03 |
| 35 | 23 | M | 20 | 74.39 | 56.90 | 1.31 | 4 | 88 | 68.35 | 52.75 | 1.30 | 1.08 |

M, male; F, female; CR, contraction ratio; MBR, muscle bulk ratio; ROM, range of motion;

CR1 was the post-transplant CR value, while CR2 was the pre- transplant CR value.
